# Single Stage Transoral Laser Microsurgery for Early Glottic Cancer

**DOI:** 10.3389/fonc.2018.00298

**Published:** 2018-08-14

**Authors:** Yaniv Hamzany, Hagit Shoffel-Havakuk, Stav Devons-Sberro, Shani Shteinberg, Dan Yaniv, Aviram Mizrachi

**Affiliations:** ^1^Department of Otolaryngology Head and Neck Surgery, Rabin Medical Center, Petach Tikva, Israel; ^2^Sackler Faculty of Medicine, Tel Aviv University, Tel Aviv, Israel; ^3^Hadassah-Hebrew University School of Medicine, Jerusalem, Israel; ^4^Institute for Speech, Swallowing and Voice Rehabilitation, Rabin Medical Center, Petach Tikva, Israel

**Keywords:** glottic cancer, CO_2_ laser, excisional biopsy, single stage, transoral laser microsurgery, complete removal

## Abstract

**Objectives:** The purpose of the study was to present the outcome of our management protocol of a single stage transoral laser microsurgery (SSTLM), with the intention of complete removal of a lesion, considered to be an early glottic cancer.

**Methods:** Between January 2015 to February 2017 patients with the clinical appearance of an early glottic cancer, who were candidates for (SSTLM) management protocol, were included in this study. Type of cordectomy was determined by pre- and intra-operative evaluation of the extent of lesion in cord layers.

**Results:** Thirty patients (6 females, 24 males; mean age 65 years) underwent SSTLM. Twenty-two patients had malignant histopathological diagnosis of severe dysplasia or Cis in 4 patients, microinvasice carcinoma in 3 patients and invasive carcinoma in 15 patients (T1a tumor in 14 and T1b tumor in 1). Eight patients had a nonmalignant histological diagnosis of keratosis without atypia in 2 patients, mild dysplasia in 2 patients and moderate dysplasia in 3 patients. Based on pre- and intra-operative evaluation, 14 subepithelial (type I), 10 subligamental (type II), and 6 transmuscular (type III) cordectomies were performed. Comparison of cordectomies types with postoperative histopathologic diagnosis showed an adequate extent of resection in 26 out of 30 patients (87%). Considering only patients without recent background of direct laryngoscopy and biopsy, an adequate resection was performed in 90% of patients. None of the patients was further treated by external beam radiation. At average follow-up of 21 months, none of the patients developed local recurrence.

**Conclusion:** In selected cases, a SSTLM for clinical appearance of an early glottic cancer, allows a reliable histopathologic diagnosis and a high local control rate with favorable cost effectiveness. A careful pre- and intraoperative evaluation for selecting the appropriate cases for this management is required in order to avoid under- or over-treatment.

## Introduction

The histological diagnosis of laryngeal epithelial lesions may range from benign (including hyperplasia, keratosis) to premalignant lesions [including dysplasia and carcinoma *in situ* (Cis)] to invasive squamous cell carcinoma (SCC) (1). Precise evaluation of suspected glottic lesion requires careful endoscopic visualization and tissue sampling. However, performing incisional biopsy, which is not representative of the entire lesion, for diagnostic purposes, has several disadvantages. While superficial biopsies, might result in inadequate sampling, or could miss the diagnosis of invasion, deep biopsies may result in inflammation and fibrotic changes at the epithelium-vocal ligament interface, these can cause an unclear borders appearance, or limit a proper evaluation of the deep extent of the residual tumor. A larger unnecessary resection might be performed in a subsequently curative procedure, potentially compromising voice outcome (2). Furthermore, in up to 33% of patients with early glottic cancer, complete tumor removal is achieved with initial biopsy, leading to unnecessary additional treatment (3, 4). Therefore, in selected cases of early glottis cancer, a careful excisional biopsy with complete removal of the entire lesion with a limited rim of healthy tissue, at the first direct laryngoscopy (DL), may provide an unequivocal diagnosis along with curative treatment and improved voice outcome.

Blakeslee et al. comprehensive report from 1984 presented the concept of CO_2_ laser single stage excisional biopsy in the selective management of T1 glottic cancer (5). Later publication by Ossoff et al., Peretti et al., Sagiston et al. and Manola et al. (2, 6–9) supported this management for Tis and T1, mostly in cases of superficial or limited lesions. Nowadays, endoscopic resection of T1 glottic carcinoma is a valid standard of care however, a review of the literature reveals a lack of recent publications discussing indications, principles and results of a single stage DL with the intention of complete removal of a lesion considered to be an early glottic cancer. This study aims to present the outcome of our management protocol of a single stage transoral laser microsurgery (SSTLM), with curative intent, for early glottic cancer.

## Materials and methods

Patients with glottic lesions, candidates for SSTLM management protocol at the department of Otolaryngology Head and Neck Surgery of Rabin Medical Center (as will be further described in detail), between January 2015 to February 2017, were included in the study. Following an approval by the institutional ethics committee the medical records of included patients were reviewed and data regarding demographics, diagnosis, pathology, outcome, voice quality, and follow-up was collected. Outcome measures included: staging at diagnosis, type of cordectomy, histopathological diagnosis, voice evaluation, further treatment and recurrence rate.

### Management protocol for SSTLM

Before being selected for this approach, all patients had to meet the following criteria.

The lesion had to have the common characteristic appearance raising a high suspicion for early glottic cancer (e.g., keratosis, irregularity, infiltration, vascularity) under careful endoscopic evaluation at the clinic by 70-degree rigid endoscope with or without videostroboscopy. The lesion had to be confined to the true vocal cords with preserved vocal cords motion. In patients with suspected deep infiltration of the vocal cord, involvement of anterior commissure, body of arytenoid, lesion extending to the superior arcuate line into the ventricle, or subglottic or supraglottic expansion, a CT scan with intravenous contrast agent was obtained. Evidence for involvement of the paraglottic fat, thyroid cartilage, body of the arytenoid, supraglottic or subglottic regions, was contraindication to SSTLM. In such cases patient underwent incisional biopsy, either by DL under general anesthesia, or by using a distal chip camera video nasolaryngoscope with a working channel under local anesthesia as an office procedure.

Patients had to be medically approved for endoscopic surgery under general anesthesia. Patients treated with antiplatelet or anticoagulation agents, were asked to discontinue the medications prior to surgery. Before signing an informed consent form, the patients received a detailed explanation about treatment options—a DL with biopsies for diagnostic purpose, being followed by a TLM or radiation therapy in case of a malignant lesion, or a SSTLM emphasizing the possibility that it might encompass an over resection influencing their voice outcome.

### Surgical technique

**Figure 1:**
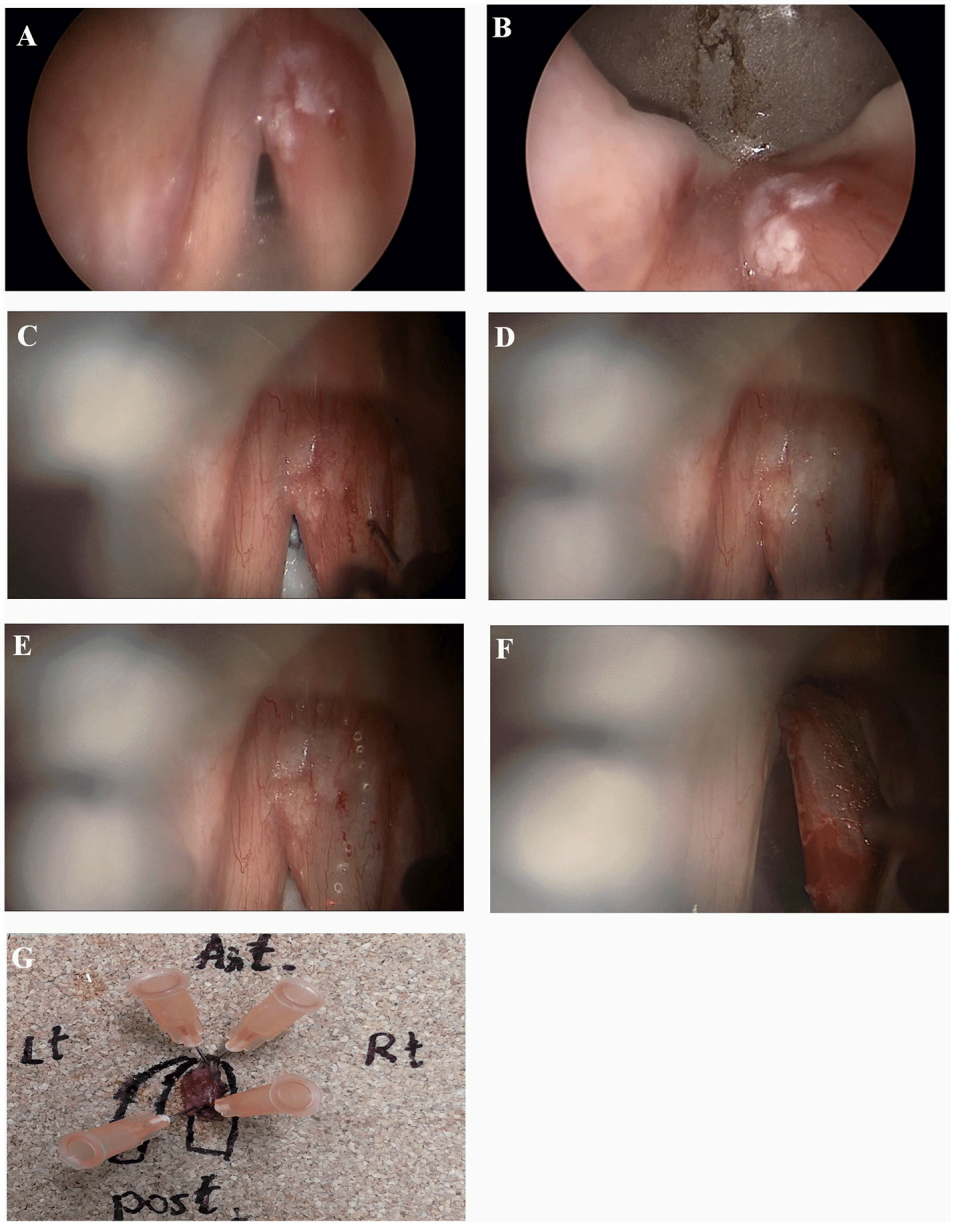
Microinvasive SCC of the right vocal cord. **(A, B)** Endoscopic view with 0 and 70-degree endoscopes. Note limited involvement of anterior commissure, no extension into ventricle or subglottic area. **(C, D)** Microscopic view before and after mucoligamentous hydrodissection by injection of saline solution into Reinke's space. Note no adherence of the mucosa to the ligament, suggesting lesion limited to the mucosa. **(E)** A narrow mucosal margin has been made with the laser. **(F)** After type I cordectomy, the deep plane of excision is the vocal ligament, which is clearly exposed. **(G)** Pathological specimen pinned on a carrier substance, oriented, and mapped by marking directions and surrounding glottic structures.

Patients were intubated using a small bore, laser safe with a double cuff, endotracheal tube (usually 5.0–6.0 mm ID). The larynx was exposed as widely as possible with suspension laryngoscopy using different shapes and sizes of laryngoscopes as needed. Counterpressure on the cricoid cartilage was performed as required for optimal visualization of the anterior commissure. Meticulous evaluation of the larynx was performed using a rigid 0 and 70-degree endoscopes (Figure 1). After achieving an adequate exposure of the glottis, microscopic examination with microinstrumantal palpation was undertaken. Surgery was performed under operating microscope, using both microsurgical instruments and CO_2_ Laser.

When needed vestibulectomy (i.e., resection of a false vocal cord or petiole of epiglottis) using CO_2_ laser was employed, in order to improve lesion exposure. After obtaining complete exposure of the lesion, including margins of healthy appearing mucosa, an adrenaline solution diluted by saline, at a concentration of 1: 100,000, was injected into Reinke's space for hydrodissection. A complete mucoligamentous hydrodissection indicated a lesion limited to the mucosa, that may be eligible for subepithelial (type I) cordectomy. If adherences were observed, indicating a lesion suspicious of involvement of the deeper layers of the vocal cord, subligamental (type II) or transmuscular (type III) cordectomy was performed corresponding with the depth of invasion as appearing under the operating microscope. CO_2_ laser resection was commenced by outlining margins of resection including a healthy mucosa of about 2 mm surrounding the lesion. Subsequently, an en-block resection was carried out in all cases. A careful clinical assessment of the margins of resection with the operating microscope and 0 and 70-degree endoscopes both during the resection and immediately on completion was done.

In case of a lesion involving both vocal cords crossing through the anterior commissure, in order to avoid post-operative anterior synechiae, a staged bilateral cordectomy was performed; the second procedure was performed after 6–8 weeks.

Cordectomy was performed using CO_2_ laser (Lumenis Laser, Yokneam, Israel) with superpulse delivery in a continuous mode (1–3 W, 270-micron spot size), coupled to an operating microscope. Transoral laser cordectomy was classified according to the European Laryngological Society (10).

### Resection margins evaluation

For histological evaluation, the surgical specimen was pinned on a carrier substance (cork board) and oriented and mapped by marking directions and surrounding glottic structures. After completion resection of the lesion, superficial and deep biopsies (sized 1–2 mm each) were obtained from the surgical bed of resection within the vocal cord (patient margins). Resection margins evaluation was based on margins at the pathological specimens and on patient margins. In case of positive margin at the specimens, but negative patient margins, patient was scheduled for close follow-up at the clinic every 1–2 months. In case of positive patient margin, additional TLM was performed with re-resection of involved margin.

### Post-operative management and follow-up

Soft oral intake was started on the operative day, and patients were discharged on the first or second postoperative day.

All patients were postoperatively examined every 2 months by a 70-degree rigid endoscope or a flexible fiberoptic endoscope. In the case of clinical suspicion of persistent or recurrent disease, a CO_2_ laser re-excision was performed.

### Voice evaluation

Patients participating in the study underwent a voice evaluation which took place at least 6 months after their last cordectomy. During voice evaluation, patients were administered with an established Hebrew version of the Voice-Related Quality of Life (V-RQOL) questionnaire (11). For perceptual voice evaluation, a standardized voice recording was performed during that visit using a head-mounted microphone placed 4 cm from the mouth. The voice recording protocol included reading of a standardized passage, sustained phonation of the vowels /a/ and /i/, load phonation and a glissando from low to high note. These voice recordings were used to determine the clinicians' perceptual voice evaluation using the GRBAS (grade, roughness, breathiness, asthenia, and strain) scale (12). A team of two clinicians experienced in voice disorders, blinded to the patient's identity, characteristics, and type of cordectomy, rated each voice sample according to the GRBAS scale. Acoustic analysis included fundamental frequency (F0), jitter, shimmer, and noise-to-harmonic ratio (NHR) was conducted with the Praat Voice Program (Paul Boersma and David Weenink, Phonetic Sciences, University of Amsterdam, Amsterdam, The Netherlands, version 6.0.36) using a sustained /a/ with a sample frequency of 44,000 Hz. Maximum phonation time (MPT) was recorded and timed during sustained /a/ phonation at a comfortable intensity and pitch level on a single breath. Patients were instructed to take a deep breath before phonating and to sustain their phonation as long as possible. The best score out of 3 trials was used for analysis.

### Data collection and analysis

Categorical variables are described by frequency and percentage; continuous variables are described by mean and standard deviation.

## Results

Between January 2015 to February 2017, 33 patients presented with clinical appearance of an early glottic cancer and were candidates for SSTLM at the Department of Otolaryngology Head and Neck Surgery of Rabin Medical Center. No initial biopsy from vocal cord was performed. Three out of 33 patients underwent DL with incisional biopsy due to inadequate exposure. Their final histopathological diagnosis was SCC and subsequently they were treated with external beam radiation therapy. Of the 30 patients who underwent SSTLM (6 females, 24 males; mean age 65; range 44–86 years), twenty-two had malignant histopathological diagnosis of severe dysplasia or Cis in 4 patients, microinvasice carcinoma in 3 patients and invasive carcinoma in 15 patients (T1a tumor in 14 and T1b tumor in 1; Table 1) (13). Five patients had anterior commissure involvement: 1, Cis; 3, T1a tumors; and 1, T1b tumor. Eight patients had a nonmalignant histological diagnosis of keratosis without atypia in 2 patients, mild dysplasia in 2 patients and moderate dysplasia in 3 patients. Based on the aforementioned criteria, 14 subepithelial (type I), 10 subligamental (type II), and 6 transmuscular (type III) cordectomies were performed. The type of cordectomy with regard to postoperative histopathological diagnosis is reported in Table 2.

**Table 1:** Clinical demographic and histological findings.

		**Malignant (*n* = 22)**		**Nonmalignant (*n* = 8)**
Gender				
Male		18		6
Female		4		2
Age				
Mean		67		60
Range		49–86		44–83
Smoking		13		6
Histological result				
	Severe dysplasia or Cis	4	Keratosis without atypia	2
	microinvasive	3	Mild dysplasia	3
	Invasive carcinoma	15	Moderate dysplasia	3

**Table 2:** Type of cordectomy according to histopathologic diagnosis.

**Histopathology**	**Patient no**.	**Type I**	**Type II**	**Type III**	**Over- treatment**
Keratosis, mild or moderate dysplasia	8	5	3	–	3
Severe dysplasia or carcinoma *in situ*	4	3	1	–	1
Microinvasive carcinoma	3	3	–	–	–
Invasive Carcinoma	15	3	6	6	–
Total	30	14	10	6	4

Staged bilateral cordectomy was performed in one patient with T1b carcinoma. One patient underwent CO_2_ laser re-excision due to superficial patient margin positive for Cis. Histopathology of re-excision showed severe dysplasia. None of the patients was further treated by external beam radiation.

The average follow-up was at 21 months, ranging from 12 to 36 months. None of the patients developed local recurrence within the follow-up period. There has been one death of unrelated cause 22 months after TLM secondary to breast cancer. There have been no intraoperative or postoperative complications in any of the patients.

Of the 22 patients with a histological diagnosis of malignancy, except one patient who died of a cause unrelated to her laryngeal disease, all completed the post-operative voice evaluation. The timing of the post-operative voice evaluation ranged from 6 to 36 months after surgery, with a median of 12 months (mean ± SD; 16.0 ± 10.2). Detailed presentation of the post-operative voice evaluation can be found on (Table 3). In general, all patients demonstrated good voice outcome results. Patients who underwent type I cordectomy had better voice outcomes than patients who underwent type II or III cordectomies, for all measures examined in our study. None of the patients had any complaints of dysphagia or sustained aspiration.

**Table 3:** Post-operative voice evaluation and acoustic analysis results.

	**Total**		**Cordectomy type I**		**Cordectomy type II**		**Cordectomy type III**	
	**(*****n*** = **21)**		**(*****n*** = **9)**		**(*****n*** = **7)**		**(*****n*** = **5)**	
**VRQOL**								
mean ± SD	16.3 ± 6.0							
median (IQR)	15 (13–18)		14 (11.5–16)		17 (10–24)		16 (15–26.5)	
**GRBAS**								
mean ± SD	18.4 ± 7.5							
median (IQR)	16 (14–21)		15 (13.5–16.5)		19 (10–28)		20 (16.5–32)	
**G**								
mean ± SD	0.71 ± 0.64							
median (IQR)	1 (0–1)		0 (0–1)		1 (0–1)		1 (1–2)	
**R**								
mean ± SD	0.25 ± 0.60							
median (IQR)	0 (0–1)		0 (0–1)		0 (0–1)		1 (0.5–1.5)	
**B**								
mean ± SD	0.24 ± 0.44							
median (IQR)	0 (0–0.5)		0 (0–0.5)		0 (0–0)		0 (0–1)	
**MPT (seconds)**								
mean ± SD	13.7 ± 7.9							
median (IQR)	12 (7.5–17)		14 (9.5–16.5)		12 (7–13)		10 (5–28.5)	
**F**_0_ **(Hz)**								
	T (*n* = 21)	M (*n* = 18)	T (*n* = 9)	M (*n* = 6)	T (*n* = 7)	M (*n* = 7)	T (*n* = 5)	M (*n* = 5)
mean ± SD	172.4 ± 83.0	171.9 ± 80.5						
median (IQR)	160 (134–194)	154.5 (134–186)	155 (134.5–194)	137 (130.5–163)	160 (133–286)	160 (133–286)	162 (116–178)	162 (116–178)
**JITTER**								
mean ± SD	1.5 ± 2.1							
median (IQR)	0.6 (0.3–1.8)		0.3 (0.28–1.6)		0.56 (0.3–3.4)		0.6 (0.39–0.92)	
**Shimmer**								
mean ± SD	7.8 ± 2.1							
median (IQR)	6.4 (4.3–8.5)		5.8 (4.2–6.9)		7.2 (6.0–11.7)		9 (5.7–13.3)	
**NHR**								
mean ± SD	0.08 ± 0.11							
median (IQR)	0.02 (0.01–0.125)		0.02 (0.001–0.025)		0.02 (0.01–0.29)		0.03 (0.02–0.13)	

*VRQOL, Voice Related Quality of Life raw (non-converted) values; SD, Standard Deviation; IQR, InterQuartile Range; G, Grade (GRBAS scale); R, roughness (GRBAS scale); B, breathiness (GRBAS scale); MPT, Maximum Phonation Time; F_0_, Fundamental frequency; NHR, Noise to Harmonic Ration; T, Total; M, Male*.

## Discussion

Incisional random biopsies, solely for diagnosis of glottic lesions, have several disadvantages. Small or superficial random biopsies can be insufficient for diagnosis or might not be taken from the malignant area of the lesion, hence leading to under diagnosis. Conversely, a more extensive biopsy, may result in complete removal of the tumor. Nassif et al. and Zapater et al. published their results on negative pathology following endoscopic resection of T1 squamous carcinoma of the glottis (3, 4). They found that following initial biopsy of an invasive SCC of the glottis, there was negative pathology in up to 33% of patients who underwent a second surgery for complete tumor resection. Thus, in a considerable number of patients the tumor is completely removed unintentionally with the initial biopsy taken, and further surgery or external beam radiation treatment is unnecessary.

SSTLM with complete removal of the entire lesion with a limited rim of healthy tissue can provide an unequivocal diagnosis along with curative treatment. Moreover, in doing so, the best compromise between oncological aspect, and voice outcome can be achieved. Performing a narrow-margin cordectomy for T1 glottic carcinoma has already been proved to be oncologically safe, provided close follow-up is undertaken, with local control rate of 93–100% at 3 years (2, 9, 14–18).

Evaluation of a glottic lesion and its borders done by DL under general anesthesia is more accurate before any biopsy is taken. Following vocal cord biopsy, the healing process causes inflammation and fibrosis which can blur the true borders between the lesion to the healthy mucosa, leading to a more extended resection during second procedure. Fibrosis and scarring of the superficial lamina propria (SLP) impairs the ability of the surgeon to assess deep involvement of the lesion beyond the mucosa. Failure to achieve a mucoligamentous separation when using the hydrodissection technique, or simply adherence of the ill mucosa to the ligament during the cordectomy, might result in resection of uninvolved deeper layers of the vocal cord.

Decision taking regarding the definitive treatment of suspected glottic lesions which solely relies on its endoscopic appearance is insufficient. Therefore, the management protocol of SSTLM requires a careful pre- and intraoperative evaluation, as described, in order to minimize the rate of under- and over- treatment cases. Peretti et al. described their experience with a pre- and intraoperative evaluation by video-laryngostroboscopy and saline infusion into Reinke's space in 52 patients with vocal cord erythroleukoplakias to predict the invasion of the layered structure of the lamina propria and consequently determine the depth of the excisional biopsy that should be carried out (19). They found that 38 patients underwent the appropriate type of resection, while 1 patient an undertreatment resection and 13 an overtreatment resection (10 of them were patients who had previous random biopsies). The authors of this publication stressed that in patients who had previous random biopsies done, the possible presence of scar tissue and fibrotic changes at the epithelium-vocal ligament interface limit the reliability of video-laryngostroboscopy and saline infusion information regarding the depth of lesion invasion. Other authors also reported that findings during video-laryngostroboscopy such as, mucosal wave absence or impaired progression correlated well with an incomplete mucoligamentous hydrodissection, thereby suggesting infiltration of the lamina propria (20, 21).

In our study, cordectomy type was mainly determined by intraoperative evaluation including hydrodissection technique and extent of tumor involvement of cord layers as observed during the resection. Comparison of cordectomies types with postoperative histopathologic diagnosis, showed a selection of the adequate treatment extent in 26 out of 30 patients (87%). None of the patients received an undertreatment. Four patients were overtreated by subligamental cordectomy (type II) while in fact could have been safely treated by subepithelial cordectomy (type I). One of these patients underwent direct laryngoscopy with vocal cord biopsies, 7 months previously, with benign histological results, possibly impairing the evaluation of the deep extent of the lesion in cord layers. Excluding this patient, an overtreatment was performed in 3 of 29 patients (10%) who underwent SSTLM, while an adequate resection was performed in 90%.

The concept of SSTLM for early glottic cancer was introduced by Blakeslee et al. as early as in the 1980s (5). They reported on 50 patients with T1 glottic cancer, of whom 46 (92%) patients were treated successfully by transoral excisional biopsy alone, thus concluding that this management can unequivocally establish the diagnosis and stage of the disease and is adequate treatment for small glottic SCC.

Peretti et al. first reported on their experience with endoscopic laser excisional biopsy for selected glottic carcinoma in 1994, limiting this treatment concept to patients without anterior commissure involvement (7). Later publication, from 2001, described their management of SSTLM in 88 patients (13 Tis, 75 T1) treated by cordectomy types I-V (2). Patients with superficial involvement of anterior commissure without cartilage invasion by CT scan were included. Five-year local control rate was 91%. Their results showed that SSTLM is an acceptable management also for infiltrative glottic cancer or cases involving the anterior commissure.

Sagiston et al. published their oncological results of laser cordectomy for early glottic cancer (8). Although detailed intraoperative evaluation and surgical technique consisted with those previously described, single stage excisional biopsies were performed only for cases of superficial lesions, by cordectomy types I-II. Their results suggest that subligamental (type II) cordectomy may be an adequate treatment for Tis or very small T1a tumors.

Manola et al. performed 29 partial cordectomies and two complete cordectomies at first operation, in 31 patients with T1 glottic carcinoma (9). Local control rate was 95%. They comment that when preoperative and intraoperative work-up is respected, the risk of under- and over-treatment is very low.

In our study, only one patient with superficial patient margin positive for Cis, underwent CO2 laser re-excision, of which histopathology showed severe dysplasia. None of the patient developed local recurrence during the follow-up period. Although our management did not include intraoperative frozen section, others have advocated the use of frozen section routinely in TLM. According to these reports, intraoperative frozen section might improve diagnostic accuracy and in some cases avoid a second procedure or the need for adjuvant radiation (22).

Our study, along with the works of other authors, shows that SSTLM can be an acceptable treatment for early glottic cancer. We argue that this management should not be limited to superficial lesions, but can also be applied in cases of infiltrative early glottic tumors or involving the anterior commissure, as was previously described by Perettei et al. and Manola et al. (2, 9).

Voice evaluation demonstrated good functional outcomes with better results in cordectomies type I and II than III. With regard to patients in other studies, with early glottic cancer who were treated by TLM after initial biopsies, voice evaluation presented equal to better voice outcomes, indicating functional benefit in cases of SSTLM (23–25).

SSTLM can also spare the burden of a second operation, which include physician, operating room and hospitalization costs, as well as patients' loss of income. If resection of the tumor has been completed at first operation, additional expenses owing to further procedures are saved.

In conclusion, SSTLM for early glottic cancer allows a reliable histopathological diagnosis and a high local control rate with favorable cost effectiveness. Moreover, doing so spares the need for further surgery which can include a more extended cordectomy along with a negative impact on voice outcome. A through patient education and counseling of the various treatment options are needed before obtaining an informed consent. A careful pre- and intraoperative evaluation for selecting the appropriate cases for this management is required in order to avoid under- or over- treatment.

## Author contributions

YH and AM designed the study. YH, DY, SD-S, and SS collected and organized the clinical data and performed the statistical analysis. YH, HS-H, and AM drafted and reviewed the manuscript. SD-S and SS revised it critically for important intellectual content. All the authors read and approved the final manuscript.

### Conflict of interest statement

The authors declare that the research was conducted in the absence of any commercial or financial relationships that could be construed as a potential conflict of interest.
